# Exploring the Potential of CRISPR-Cas9 Under Challenging Conditions: Facing High-Copy Plasmids and Counteracting Beta-Lactam Resistance in Clinical Strains of *Enterobacteriaceae*

**DOI:** 10.3389/fmicb.2020.00578

**Published:** 2020-04-30

**Authors:** Thaysa Leite Tagliaferri, Natália Rocha Guimarães, Marcella de Paula Martins Pereira, Liza Figueiredo Felicori Vilela, Hans-Peter Horz, Simone Gonçalves dos Santos, Tiago Antônio de Oliveira Mendes

**Affiliations:** ^1^Department of Microbiology, Institute of Biological Sciences, Universidade Federal de Minas Gerais, Belo Horizonte, Brazil; ^2^Institute of Medical Microbiology, RWTH Aachen University Hospital, Aachen, Germany; ^3^Department of Biochemistry and Immunology, Institute of Biological Sciences, Universidade Federal de Minas Gerais, Belo Horizonte, Brazil; ^4^Department of Biochemistry and Molecular Biology, Universidade Federal de Viçosa, Viçosa, Brazil

**Keywords:** CRISPR-Cas9, antimicrobial resistance, *bla*_TEM_, re-sensitization, resistance reduction, plasmid maintenance

## Abstract

The antimicrobial resistance (AMR) crisis urgently requires countermeasures for reducing the dissemination of plasmid-borne resistance genes. Of particular concern are opportunistic pathogens of *Enterobacteriaceae*. One innovative approach is the CRISPR-Cas9 system which has recently been used for plasmid curing in defined strains of *Escherichia coli*. Here we exploited this system further under challenging conditions: by targeting the *bla*_TEM–__1_ AMR gene located on a high-copy plasmid (i.e., 100–300 copies/cell) and by directly tackling *bla*_TEM–__1_-positive clinical isolates. Upon CRISPR-Cas9 insertion into a model strain of *E. coli* harboring *bla*_TEM–__1_ on the plasmid pSB1A2, the plasmid number and, accordingly, the *bla*_TEM–__1_ gene expression decreased but did not become extinct in a subpopulation of CRISPR-Cas9 treated bacteria. Sequence alterations in *bla*_TEM–__1_ were observed, likely resulting in a dysfunction of the gene product. As a consequence, a full reversal to an antibiotic sensitive phenotype was achieved, despite plasmid maintenance. In a clinical isolate of *E. coli*, plasmid clearance and simultaneous re-sensitization to five beta-lactams was possible. Reusability of antibiotics could be confirmed by rescuing larvae of *Galleria mellonella* infected with CRISPR-Cas9-treated *E. coli*, as opposed to infection with the unmodified clinical isolate. The drug sensitivity levels could also be increased in a clinical isolate of *Enterobacter hormaechei* and to a lesser extent in *Klebsiella variicola*, both of which harbored additional resistance genes affecting beta-lactams. The data show that targeting drug resistance genes is encouraging even when facing high-copy plasmids. In clinical isolates, the simultaneous interference with multiple genes mediating overlapping drug resistance might be the clue for successful phenotype reversal.

## Introduction

Antimicrobial resistant microorganisms have become a public health concern due to their impact on human morbidity and mortality in recent decades. The reality of ineffective drugs allied to the insufficient launching of new antimicrobials may result in about 10 million deaths caused by multidrug-resistant organism infections in 2050 (O’Neil, 2016). The costs involved in the development of a new drug amount to around 800 million dollars and it can take up to 12 years until its commercialization (Adams and Brantner, 2006; Van Norman, 2016). Of particular concern is antimicrobial resistance (AMR) evolving in and spreading across species from the *Enterobacteriaceae* family, which includes plasmid encoded extended-spectrum beta-lactamases and carbapenemases as the main mechanism to disrupt antimicrobials (Savard and Perl, 2012).

Plasmids are well known for facilitating bacterial adaptation by their vertical and horizontal transmission. More specifically, small (<10 kb) multicopy plasmids have an important role in counteracting antimicrobial stressors. Multiple copies of a resistance gene increase the probability of mutational adaptation, thereby rapidly generating allele variations (Hall and Harrison, 2016). In addition, the repeated exposure to beta-lactams and high levels of beta-lactam resistance have been shown to be associated with an increase of plasmid copy number, reaching values higher than 100 copies/cell (San Millan et al., 2015, 2016). Hence, the increase of plasmid copy numbers can lead to maximum levels of AMR (San Millan et al., 2015, 2016; Hall and Harrison, 2016; Schechter et al., 2018).

Considering the capacity of plasmid-based resistance dispersal and the global public health impact of resistant bacteria, studies are needed for developing new technologies that could impede this progression. In recent years, CRISPR-Cas9 [clustered regularly interspaced short palindromic repeats (CRISPR)-CRISPR-associated protein 9 (Cas9)] has been demonstrated as an effective tool to cleave double-stranded DNA with accuracy (Ceasar et al., 2016). First studies have already successfully employed the CRISPR-Cas technology for genetically targeting AMR in different bacteria (Bikard et al., 2014; Citorik et al., 2014; Yosef et al., 2015). For instance, Yosef et al. (2015), not only reverted AMR but also eliminated the transfer of plasmids encoding two different beta-lactamases among *Escherichia coli* strains. Bikard et al. (2014) and Citorik et al. (2014) achieved plasmid curing and observed a CRISPR-Cas9 mediated cytotoxicity after specifically editing AMR genes in *Staphylococcus aureus* and in *E. coli*, respectively.

Given the importance of plasmid copy number variation in a cell (San Millan et al., 2015) and the overall complexity of AMR mechanisms within clinical isolates (Blair et al., 2015) we here investigated the potential of the CRISPR-Cas9 system under two challenging conditions. First, the CRISPR-Cas9 system was used to specifically target the *bla*_TEM__–__1_ gene, located on the small high-copy plasmid pSB1A2, with 100–300 copies/cell (Yang et al., 2013; Registry of Standard Biological Parts, 2019), using a model *E. coli* strain. The *bla*_TEM_ gene codifies for one of the most frequently encountered beta-lactamase in *Enterobacteriaceae* (Lachmayr et al., 2009; Rawat and Nair, 2010). Subsequently, we investigated the re-sensitization effect when targeting the same gene in clinical isolates of *E. coli* and related *Enterobacteriaceae* species.

## Materials and Methods

### *Escherichia coli* Model Strain and Plasmids Used

The *E. coli* strain BL21 (Studier and Moffatt, 1986; Kim et al., 2017) was used in this study to validate the CRISPR-Cas9 system when targeting a high-copy plasmid. Bacteria were cultivated in LB medium at 37°C and supplemented with ampicillin, 100 μg/ml; chloramphenicol, 30 μg/ml; and kanamycin, 50 μg/ml, depending on the plasmids harbored by the strains (Table 1). Three plasmids were used in this study, pSB1C3, pSB1A2, and pSB1K3, which are available at the Registry of Standard Biological Parts^1^ under the accession numbers BBa_K1218011, BBa_J04450, and BBa_I20260, respectively.

**TABLE 1:** Plasmids used in this study, their relevant features and associated strains.

**Plasmid features**			
**Plasmid name**	**Size**	**Relevant features**	**References**
pSB1A2	3148 bp	Amp^R^ mediated via *bla*_TEM–1_ gene (sgRNA target); red fluorescent protein (RFP) gene.	(Yang et al., 2013; Registry of Standard Biological Parts, 2019)
pSB1K3	3123 bp	Km^R^; green fluorescent protein (GFP) gene.	(Registry of Standard Biological Parts, 2019)
pSB1C3	7150 bp	Cm^R^; contains the *Streptococcus pyogenes* CRISPR-Cas9 loci (sgRNA targeting the *bla*_TEM–1_ gene was added in this study).	(Registry of Standard Biological Parts, 2019)

***E. coli* BL21 strains containing the plasmids**			
**Strain number**	**Name**	**Description**	**References**

1	BL21^–^	*E. coli* BL21	(Studier and Moffatt, 1986; Kim et al., 2017)
2	CRISPR^+^	*E. coli* BL21 with the plasmid pSB1C3	This study
3	TEM^+^	*E. coli* BL21 with the plasmids pSB1A2 and pSB1K3	This study
4	TEM^+^/CRISPR^+^	*E. coli* BL21 with the plasmids pSB1A2, pSB1K3 and pSB1C3	This study

*Cm^*R*^, chloramphenicol resistance as selective marker; Amp^*R*^, ampicillin resistance as selective marker; Km^*R*^, kanamycin resistance as selective marker.*

The pSB1C3 plasmid contains a constitutively expressed *Streptococcus pyogenes* derived CRISPR-Cas9 system. The sequence of the sgRNA responsible for the specificity of Cas9 cleavage was designed by indoor scripts using Pearl language based on public available sequences of the 861 bp-long *bla*_TEM–__1_ gene and its variants *bla*_TEM–__1__a__–__d_ (Supplementary Figure S1A), including members of the *Enterobacteriaceae*. The sliding window method was used to find all 20-nucleotide length sequences with NGG flanking the 3-prime end. The conserved region most closely located at the 5′-end of the gene was selected (AGATCAGTTGGGTGCACGAGTGG). After synthesis of the 5′-located sgRNA, it was phosphorylated using a polynucleotide kinase (Thermo Fischer Scientific, MA, United States) and inserted into the plasmid pSB1C3 containing the CRISPR-Cas9 locus.

### Clinical Isolates

*Escherichia coli* 189A, *Enterobacter hormaechei* 4962 and *Klebsiella variicola* 68AI were isolated from patients with bacteremia in two different hospitals of Belo Horizonte, Brazil (Supplementary Table S2). Clinical strain collection was approved by the Human Research Ethics Committee from Universidade Federal de Minas Gerais (Brazil) under the protocol number ETIC 614/08 and written informed consent was obtained from each participant. *E. hormaechei* belongs to the *Enterobacter cloacae* complex and *K. variicola* is a member of the *Klebsiella pneumoniae* complex, as they share some biochemical and phenotypical features with *E. cloacae* and *K. pneumoniae*, respectively (Hoffmann et al., 2005; Barrios-Camacho et al., 2019). Because of this similarity, the species are frequently misclassified and the worldwide presence of these bacteria in human infections may be underestimated, although their relevance as clinical pathogens has been demonstrated (Long et al., 2017; Beyrouthy et al., 2018; Barrios-Camacho et al., 2019; Rodríguez-Medina et al., 2019). The selection criteria of the isolates were the absence of a natural chloramphenicol-resistant phenotype, since the antibiotic was used as a selective marker of the pSB1C3 plasmid; the presence of the *bla*_TEM–__1_ gene and the absence of carbapenemase genes.

### Plasmid Transformation

Chemically competent *E. coli* BL21 were prepared using 0.1M MgCl_2_–CaCl_2_ and competent cells were then transformed with plasmids by the heat-shock method (Chan et al., 2013; Lim et al., 2015). The clinical isolates used in this study received the CRISPR-Cas9 plasmid pSB1C3 by electroporation (Gonzales et al., 2013).

### Polymerase Chain Reactions

All primers used for polymerase chain reactions (PCRs), as well as the reaction conditions are provided in Supplementary Table S1. Cas9 functionality and *bla*_TEM–__1_ expression were assessed by reverse transcription quantitative polymerase chain reaction (RT-qPCR), which were performed based on cDNA from reverse transcription of total RNA. The 16S rRNA served as endogenous transcript for an internal control. Total bacterial RNA was extracted using TRIzol reagent and DNA was removed using DNase (both from Thermo Fischer Scientific, MA, United States). Integrity of extracted RNA was evaluated by agarose gel electrophoresis. Reverse transcription was performed with 1 μg of total RNA according to the iScript cDNA Synthesis Kit protocol (Bio-Rad, CA, United States). RT-qPCR was performed based on the CFX 96 Real Time PCR Detection System using the SYBR Green Master Mix (both from Bio-Rad, CA, United States). The analyses were performed by the relative standard curve method using a serial dilution of total cDNA pool as previously described (Larionov et al., 2005).

For determination of total plasmid copy number, the *bla*_TEM–__1_ gene was selected as a plasmid target. The amplified products of the chromosomal 16S rRNA gene and the *bla*_TEM–__1_ gene were cloned into TOPO vector (Thermo Fischer Scientific, MA, United States) for subsequent construction of the standard curves and absolute quantification. Determination of bacterial cell numbers was based on 16S rRNA gene quantification under consideration of the average 16S copy numbers per cell in strains (Klappenbach et al., 2000; Větrovský and Baldrian, 2013). Establishment of standards with defined amounts of DNA was performed as previously described (Vianna et al., 2008).

### Growth Curves

Overnight bacterial cultures were adjusted to an OD_600 nm_ of 0.1 and re-grown in LB medium with and without ampicillin. The growth kinetics were recorded every hour for 24 h similarly described in Jansen et al. (2018). Each assay was performed in triplicate and the whole experiment, starting from the pSB1C3 transformation, was performed three times on different days, accounting for technical and biological replicates.

### Disk Diffusion Test and Minimum Inhibitory Concentration

The disk diffusion test was performed in duplicate according to the Change to Clinical and Laboratory Standards Institute and the European Committee on Antimicrobial Susceptibility Testing (CLSI) protocol (Clinical and Laboratory Standards Institute, 2019). In total, 12 antibiotics were selected, namely chloramphenicol (CHL), ampicillin (AMP), ampicillin/sulbactam (SAM), amoxicillin/clavulanic acid (AMC), cefazolin (CFZ), cefoxitin, cefuroxime (CXM), ceftriaxone (CRO), ceftazidime (CAZ), cefotaxime (CTX), cefepime (FEP), and aztreonam (ATM). Measurements of diameter of the inhibition zones were performed in duplicate. For the *Galleria mellonella* assay, the minimum inhibitory concentration (MIC) of the clinical isolate of *E. coli* 189A was determined using the microdilution protocol (Clinical and Laboratory Standards Institute and Weinstein, 2012).

### Fluorescence Measurement

Fluorescence microscopy was performed using the EVOS^®^ FL microscope (Life Technology, CA, United States). Cells were analyzed at the bright field and with the green fluorescent protein (GFP) (ex:470 nm/em:524 nm) and red fluorescence protein (RFP) (ex:530 nm/em:593 nm) fixed filters. For an overall analysis of RFP and GFP intensity, fluorescence was quantified using the Cytation 5 Cell Imaging Multi-Mode Reader (BioTeck, VT, United States). Fluorescence experiments were performed in triplicate and the whole experiment, starting from the pSB1C3 transformation, was performed three times on different days, accounting for technical and biological replicates.

Fluorescence-activated cell sorting (FACS) (BD FACS Canto II, BD, NJ, United States) was employed to distinguish between RFP positives and negative cells upon CRISPR-Cas9 treatment. Each measurement analyzed 30,000 events at a low flow rate. The following settings were used: an SSC voltage of 473; a FSC voltage of 398; and a PerCP-Cy5-5-A of 445 V.

### Sequencing Analyses

Sanger sequencing of purified PCR products was performed by Myleus Biotechnology (Minas Gerais, Brazil) and Eurofins Genomics (Luxembourg, Luxembourg). Sanger sequence analyses were performed using the Phred/Phrap pipeline and the Degenerate Sequence Decode program (DSDecodeM) (Liu et al., 2015). Deep sequencing of the genomes was performed using the Miseq platform (Illumina, CA, United States). Reads were trimmed to Phred15 using the Trimmomatic program (Bolger et al., 2014). The *de novo* assembly of the contigs was performed via the St. Petersburg genome assembler (SPAdes) (Bankevich et al., 2012) and resistance genes were detected via ResFinder (Zankari et al., 2012). Genome sequences were submitted to GenBank (accession number: *E. coli* SAMN12872130, *E. hormaechei* SAMN12872875 and *K. variicola* SAMN10216245.

### *Galleria mellonella* Infection Model

Larvae of the great wax moth *G. mellonella* were selected according to their length (2–3 cm, instar stage), weight (150–250 mg), and excluded in case of dark coloration or limited activity. They were subsequently placed in the dark without food supply for 24-h acclimation prior to the infection assay (Harding et al., 2013). Larvae were challenged with *E. coli* 189A treated or untreated with CRISPR-Cas9 (∼4.0 × 10^6^ CFU/ml). After 1-h of infection, either ceftriaxone (16 mg/kg) or sterile distilled water was administered in the front larvae proleg with a 10 μl syringe (Hamilton, NV, United States). Each group contained ten larvae sorted randomly, and the experiment was performed in triplicate (*n* = 30). Larvae were incubated at 37°C, monitored after 24, 48, and 72 h and death was determined by absence of movement and unresponsiveness to touch.

### Statistical Analysis

Statistical analyses were performed using the GraphPad Prism (version 6, GraphPad Software, CA, United States), the VassarStats (NY, United States), as well as the BD FACSDiva and FlowJo softwares.

## Results

### Resistance Reversal in a Model Strain of *E. coli*

Initially, the *bla*_TEM__–__1_ gene located on the small high-copy plasmid pSB1A2 was introduced into the *E. coli* strain BL21, which was otherwise devoid of AMR. Located on this plasmid was also the RFP gene, as a marker, to verify the integrity of pSB1A2 upon CRISPR-Cas9 insertion. A second plasmid, pSB1K3, which contained the GFP gene, but no CRISPR-Cas9 target was also introduced into BL21. This plasmid served as an independent and indirect measurement of plasmid stability. The two plasmids pSB1A2 and pSB1K3 were chosen because of their high-copy number (i.e., 100–300 copies/cell) (Yang et al., 2013; Registry of Standard Biological Parts, 2019). For the design of a proper sgRNA, the conserved region most closely located at the 5′-end of the *bla*_TEM__–__1_ gene was selected (Supplementary Figure S1A) in order to maximize the likelihood of an early stop codon generated during an eventual bacterial DNA repair mechanism (Chayot et al., 2010). Basic features of the three plasmids used in this study and associated strains are given in Table 1.

**FIGURE 1:**
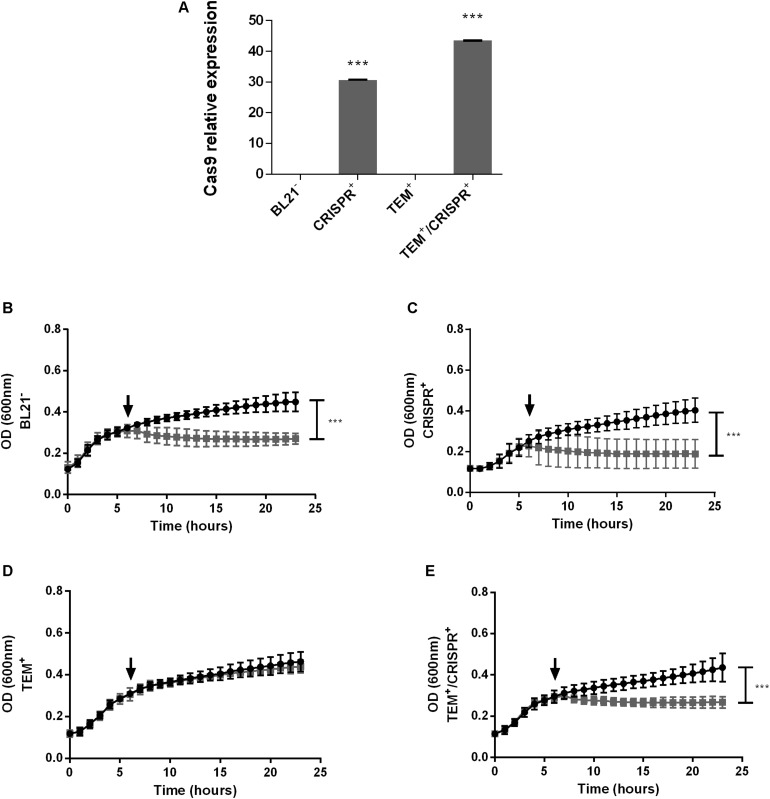
CRISPR-Cas9 expression and growth curves of an *E. coli* model strain. **(A)** Relative expression of Cas9 in *E. coli* BL21; BL21^–^: BL21 strain with no plasmid, CRISPR^+^: strain containing the CRISPR-Cas9 system, TEM^+^: strain containing the *bla*_TEM–__1_ resistance gene, TEM^+^/CRISPR^+^: strain containing *bla*_TEM–__1_ and CRISPR-Cas9. Normalization was performed based on the 16S rRNA housekeeping gene. Bars represent the mean of two real-time quantitative PCR assays ± standard deviation. **(B–E)** Growth of *E. coli* BL21 in the presence (gray curve) or absence (black curve) of ampicillin. **(B)** BL21^–^; **(C)** CRISPR^+^; **(D)** TEM^+^; **(E)** TEM^+^/CRISPR^+^. The time point of ampicillin addition is indicated by a black arrow. Each curve represents the mean of three biological and three technical replicates ± standard deviation. Stars indicate statistical support for differences between strains with or without the CRISPR-Cas9 system, ***p < 0.001.

The presence and expression of Cas9 could be confirmed in CRISPR^+^ and TEM^+^/CRISPR^+^, but not in BL21^–^ or TEM^+^ (Figure 1A), as expected. Growth curve analyses in the presence or absence of ampicillin confirmed antibiotic sensitivity of BL21^–^ and CRISPR^+^ (Figures 1B,C, respectively). In contrast, TEM^+^ grew in the presence of ampicillin demonstrating the effectiveness of the introduced beta-lactamase gene (Figure 1D). The targeted re-sensitization to ampicillin was achieved in TEM^+^/CRISPR^+^ (Figure 1E). Disk diffusion tests confirmed resistance of TEM^+^ and sensitivity of TEM^+^/CRISPR^+^ strains (Supplementary Figure S1B).

**FIGURE 2:**
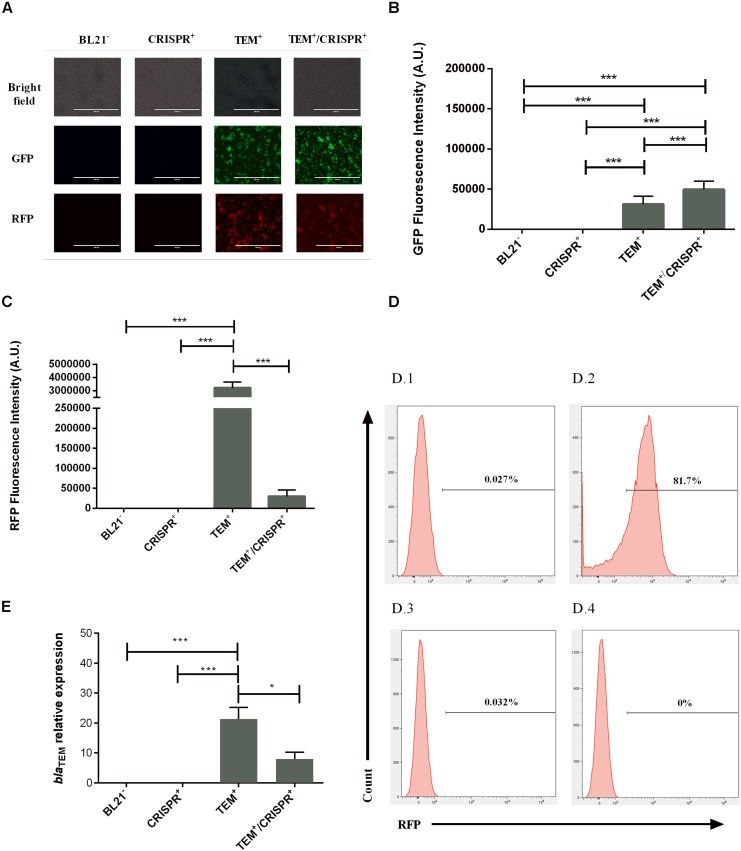
Effect of the CRISPR-Cas9 transformation on plasmid maintenance and *bla*_TEM–__1_ gene expression in *E. coli* BL21; BL21^–^: BL21 strain with no plasmid, CRISPR^+^: strain containing the CRISPR-Cas9 system, TEM^+^: strain containing the *bla*_TEM–__1_ resistance gene, TEM^+^/CRISPR^+^: strain containing *bla*_TEM–__1_ and CRISPR-Cas9 **(A)** Fluorescence microscopy indicating bacterial viability and maintenance of the plasmids pSB1A2 and pSB1K3 based on the RFP/GFP signals. For each strain, pictures were taken at the same microscopy field (40× objective). **(B)** GFP fluorescence intensity indicating the continued presence of plasmid pSB1K3 (thus no natural causes of plasmid reduction) in strain TEM^+^/CRISPR^+^. A.U., arbitrary units. **(C)** RFP fluorescence intensity indicating a significant reduction of the plasmid pSB1A2 (but no entire plasmid loss) in strain TEM^+^/CRISPR^+^ compared to TEM^+^. Bars represent the mean of three biological and three technical replicates ± standard deviation. A.U., arbitrary units. **(D)** Histograms of negative (D.1) and the positive controls (D.2) of FACS analysis used to demarcate the gate areas. The plot (D.3) represents the colony with higher RFP percentage (0.032%), while (D.4) demonstrates the colony with no RFP signal (0%). When analyzing all retrieved colonies, 0.005% of RFP positive cells were present upon CRISPR-Cas9 insertion. A control experiment with the pSB1C3 vector lacking the sgRNA led to a high percentage of RFP-positive cells (85.2%), similar to TEM^+^ cells, confirming the sgRNA-dependant plasmid reduction. **(E)** Relative expression of the *bla*_TEM–__1_ gene confirming plasmid presence and gene functioning in the cells. Bars represent average and standard deviation of two replicate assays. The results were normalized with the 16S rRNA housekeeping gene. *p < 0.05; ***p < 0.001.

We observed a 100-fold lower RFP signal in TEM^+^/CRISPR^+^, compared to TEM^+^ (*p* < 0.001) indicating a strong but not entire reduction of pSB1A2 (Figures 2A,C) upon CRISPR-Cas9 insertion. FACS results confirmed that only 0.005% of cells were RFP positive (Figures 2D, plot D.1 and D.3), which represents an around 150-fold reduction compared to the TEM^+^ cells (Figure 2D, plot D.2). A control experiment with the pSB1C3 vector lacking the sgRNA led to a high percentage of RFP-positive cells (85.2%), similar to TEM^+^ cells, confirming that the plasmid reduction was due to the presence of the sgRNA. This result combined with quantitative PCR analysis (qPCR) of the *bla*_TEM_ gene indicated, that on average, the 0.005% persistent RFP positive TEM^+^/CRISPR^+^ cells carried around 48 pSB1A2 copies/cell. In contrast, TEM^+^ cells harbored around 100 copies/cell of pSB1A2. Since the GFP signals did not decrease (Figures 2A,B), pSB1A2 reduction was the result of CRISPR-Cas9 activity rather than any unspecific plasmid clearance by the cell.

In keeping with the RFP and plasmid reduction, the expression of the *bla*_TEM__–__1_ gene was significantly reduced (by more than 2.5-fold, *p* < 0.05) in TEM^+^/CRISPR^+^, again confirming reduction but not entire plasmid loss (Figure 2E). Since those persistent gene expression levels did not confer ampicillin resistance (Figure 1E and Supplementary Figure S1B), it is likely that gene defects occurred. In fact, deletions could be seen in the sequence data of the *bla*_TEM__–__1_ resistance gene (Supplementary Figure S2A). This means, that any possible transmission of the remaining plasmid, vertically or horizontally, would not necessarily lead to the spread of *bla*_TEM–__1_-based AMR, because of the non-functional resistance gene.

However, cases where the CRISPR-Cas9 was able to completely eradicate the high-copy plasmid pSB1A2 were also achieved, as evident by FACS analysis (Figures 2D, plot D.4) and confirmed by qPCR.

### Resistance Reversal in the Clinical Isolate of *E. coli* 189A

**FIGURE 3:**
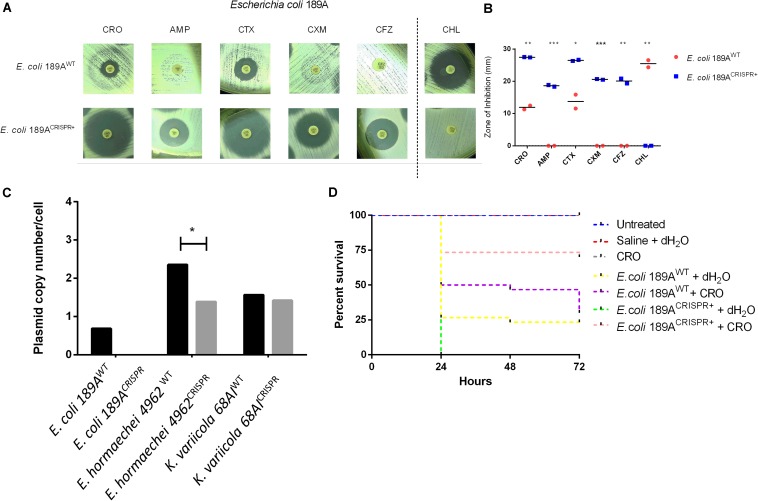
Effect of the CRISPR-Cas9-based interference with the *bla*_TEM–__1_ gene in the clinical isolate of *E. coli* 189A. **(A,B)** Disk diffusion susceptibility test on *E. coli* grown on Mueller-Hinton agar with measurement of inhibition zones showing re-sensitization to ceftriaxone (CRO), ampicillin (AMP), cefotaxime (CTX), cefuroxime (CXM), and cefazolin (CFZ), along with acquired resistance against chloramphenicol (CHL), co-mediated via the CRISPR-Cas9 plasmid. ^WT^, wild type; ^CRISPR+^, presence of the CRISPR-Cas9 plasmid. Horizontal bars represent the mean of duplicate disk diffusion tests. *p < 0.05; **p < 0.01; ***p < 0.001. **(C)** Quantification of the *bla*_TEM–__1_ plasmid in clinical isolates of *Enterobacteriaceae* before and after CRISPR-Cas9 treatment. qPCR-based amplification of the *bla*_TEM–__1_ gene indicated plasmid clearance in *E. coli* 189^CRISPR+^ and plasmid maintenance in *E. hormaechei* 4962^CRISPR+^ and *K. variicola* 68AI^CRISPR+^. **(D)** Percent survival rate of *G. mellonella* larvae after infection with either *E. coli* 189A^WT^ or *E. coli* 189A^CRISPR+^ plus administration of ceftriaxone (CRO) or water 1 h post-infection. A higher proportion of larvae survived when infected with *E. coli* 189A^CRISPR+^ as opposed to those infected with *E. coli* 189A^*WT*^ (*p* < 0.01), indicating re-usability of CRO upon treatment with CRISPR-Cas9-system. The data are the mean of three independent experiments, each performed with 10 larvae per treatment group (*n* = 30).

We next tested a *bla*_TEM–__1_ positive clinical isolate of *E. coli* 189A. Principally, considering that *bla*_TEM_ variants potentially confer resistance to many beta-lactam antibiotics (Rawat and Nair, 2010), targeting this gene might restore the usability of several antibiotics. In fact, after CRISPR-Cas9 insertion into the clinical isolate of *E. coli* 189A, a re-sensitization was observed to AMP, CFZ, CXM, CRO, and CTX (Figures 3A,B and Supplementary Figure S2B), along with an introduced resistance against chloramphenicol co-mediated via pSB1C3. Based on qPCR, the *bla*_TEM__–__1_ harboring plasmid was found to occur in low-copy numbers, as on average less than a single copy per bacterial cell was detected, indicating that some bacteria might be already plasmid free. After the CRISPR-Cas9 insertion, the *bla*_TEM_ gene was not detectable anymore, indicating complete eradication of the plasmid (Figure 3C). The achieved plasmid clearance also resulted in the extinction of other resistance genes conferring resistance to beta-lactams and other classes of antibiotics. Genome sequencing identified the *sul*2 (sulfonamide), *aph*(3″)-Ib and *aph*(6)-Id (both confer resistance to aminoglycoside), as well as *bla*_CTX–M–__9_ (beta-lactam) genes before, but not after the CRISPR-Cas9 insertion.

To further investigate the meaningfulness of the re-sensitization approach, the reusability of CRO as one representative antibiotic was verified by infecting larvae of the great wax moth *G. mellonella* either with the CRISPR-Cas9 treated *E. coli* 189A (*E. coli* 189A^CRISPR+^) or with the *E. coli* 189A wild type (*E. coli* 189A^WT^).

Upon administration of CRO, 70% of larvae infected with *E. coli* 189A^CRISPR+^ survived the time period of 72 h, as opposed to only 30% of larvae infected with *E. coli* 189A^WT^ (log-rank test, *p* < 0.01) (Figure 3D). No significant difference was seen between the CRO-treated larvae challenged with *E. coli* 189A^CRISPR+^ and larvae used as controls (i.e., mock-infection with saline or administration of antibiotic only, or no treatment at all. Log-rank test, *p* > 0.05). Conversely, it made no difference whether *E. coli* 189A^WT^ infected larvae were treated with antibiotic or with water (log-rank test, *p* > 0.3) (Figure 3D). The outcome of this experiment indicates that antibiotic-treated larvae had a 5.44 times higher chance of survival, when challenged with *E. coli* 189A^CRISPR+^ rather than with *E. coli* 189A^WT^ (*p* < 0.01).

### Resistance Reduction in Clinical Isolates of *E. hormaechei* 4962 and *K. variicola* 68AI

**FIGURE 4:**
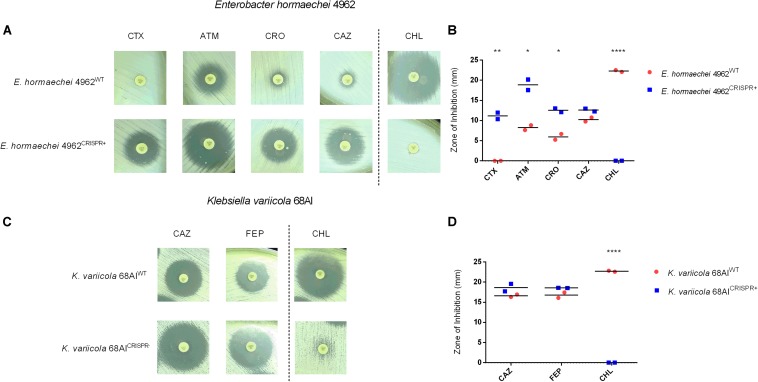
Effect of the CRISPR-Cas9-based interference with the *bla*_TEM–__1_ gene in a clinical isolate of *E. hormaechei* 4962 and *K. variicola* 68AI. **(A,B)** Disk diffusion susceptibility test on *E. hormaechei* 4962 grown on Mueller-Hinton agar with measurement of inhibition zones. The level of resistance could be significantly reduced for aztreonam (ATM), cefotaxime (CTX), and ceftriaxone (CRO) along with acquired resistance against chloramphenicol (CHL). Note that only for ATM the intermediate sensitivity category was reached according to CLSI-definitions. **(C,D)** Disk diffusion susceptibility test on *K. variicola* 68AI grown on Mueller-Hinton agar with measurement of inhibition zones showing minor resistance reductions for ceftazidime (CAZ) and cefepime (FEP), along with acquirement of full resistance against CHL. Horizontal bars represent the mean of duplicate disk diffusion tests. *p < 0.05; **p < 0.01; ****p < 0.0001.

A more complex situation was encountered when targeting the *bla*_TEM–__1_ gene of *E. hormaechei* 4962 *and K. variicola* 68AI. Upon CRISPR-Cas9 insertion, significant increases in the inhibition zones were observed for ATM, CTX, and CRO in the case of *E. hormaechei* (Figures 4A,B and Supplementary Figure S2B). However, the CLSI-defined threshold levels for an intermediate sensitivity was only reached for ATM. In some disk diffusion assays, a few colonies grew within the inhibition zones which were, however, not further analyzed (Figure 4A). For *K. variicola*, only non-significant increases in the inhibition zones were observed for CAZ and FEP (Figures 4C,D and Supplementary Figure S2B).

Further analysis revealed the continued presence of the *bla*_TEM__–__1_ gene in *E. hormaechei* 4962 and *K. variicola* 68AI, in contrast to what was observed with the clinical isolate of *E. coli* 189A, which could be re-sensitized for several antibiotics. qPCR-based detection of the *bla*_TEM–__1_ gene showed that a substantial plasmid reduction had occurred in *E. hormaechei* 4962, as opposed to only a negligible fraction of plasmid reduction in *K. variicola* 68AI (Figure 3C). Since both *E. hormaechei* 4962 and *K. variicola* 68AI were found to harbor the *bla*_TEM–__1_ gene on a low-copy number plasmid (Figure 3C), we expected to achieve plasmid clearance. However, damages on the CRISPR-Cas9 loci after insertion were observed in both strains, affecting the expected CRISPR-Cas9-based outcome (Supplementary Figure S3). These findings explain the lower efficiency of re-sensitization when compared to the clinical strain of *E. coli* 189A.

Importantly, other genes conferring resistance to beta-lactams could be identified by genome sequencing in both strains. In *E. hormaechei* 4962, the intrinsic AmpC-type resistance gene was present (*bla*_ACT–__7_), as well as the beta-lactamases *bla*_CTX–M–__9_ and *bla*_OXA–__9_. The same *bla*_CTX–M–__9_ was also detected in *K. variicola* 68AI, along with the *bla*_LEN__16_ and *bla*_LEN__19_ genes conferring resistance to beta-lactams. Since these resistance genes were not targeted by the sgRNA, failure to achieve fully reverted phenotypes was likely due to their presence in the bacteria, in addition to the damages in the CRISPR-Cas9 plasmid region.

## Discussion

In this study, we investigated the potential of the CRISPR-Cas9 system to counteract antibiotic resistance mediated by the *bla*_TEM–__1_ gene harbored either in the high-copy plasmid pSB1A2 or when present in clinical isolates. In a model strain of *E. coli* having received the *bla*_TEM–__1_ gene on a high-copy plasmid (i.e., 100–300 copies/cell) the phenotype could be clearly reversed. However, an entire elimination of the plasmid and the target gene did not occur in some retrieved colonies. This outcome is likely linked with the initial high overall abundance of the vector, since CRISPR-Cas9 is able to completely clear a resistance gene plasmid, when the copy number per cell ranges between 50 and 70 (Citorik et al., 2014). Those remaining plasmid-positive cells, could be considered “persister cells” in an analogy to bacteria persisting antibiotic treatment (Van den Bergh et al., 2017), given their low fraction within the whole population, and their endurance in the presence of CRISPR-Cas9 concomitant with an otherwise re-sensitized phenotype (Wood et al., 2013; Van den Bergh et al., 2017). However, even facing high-copy plasmids, CRISPR-Cas9 demonstrated the potential to completely clear the vector from some bacterial colonies, indicating its promising application under this challenging condition. Optimization of the system may be required for a complete clearance of high-copy plasmids carrying resistance genes in the whole population.

The reason why a bacterial subpopulation maintains the plasmid while being attacked by CRISPR-Cas9 remains unknown. However, laboratorial and pathogenic *E. coli* strains possess an end-joining repair mechanism to bridge broken DNA ends (Chayot et al., 2010). Named as alternative end-joining (A-EJ), this restoration attempt typically leads to microdeletions in the sgRNA region (affecting regions of 1–10 bp), similar to those observed in this study (Supplementary Figure S2A; Chayot et al., 2010; Chen et al., 2019). Although the deletions lead to a non-functional *bla*_TEM–__1_ gene, the possible existence of CRISPR-Cas9-persisters in the context of clinical isolates, could still result in therapeutic failure, because other intact resistance genes might still reside on the plasmid. Therefore, the detection and knowledge of CRISPR-Cas9-persisters may be important for devising strategies to minimize their potential clinical impact.

The deleterious effects on plasmids and bacterial cells of CRISPR-Cas9 targeting other resistance genes have been previously reported. Bikard et al. (2014) interfered with the kanamycin and the methicillin resistant genes *aph-3* and *mecA*, in *S. aureus* (MRSA), while Citorik et al. (2014) re-sensitized *E. coli* that possessed the *bla_NDM–1_* and *bla*_SHV–__18_ genes. In both studies, a cytotoxicity of the CRISPR-Cas9 system was apparent with episomal and chromosomal targets. In the first situation, bacteria were killed because plasmid clearance led to the loss of the toxin-antitoxin genes. As the antitoxin is less stable than the toxin, its faster degradation results in cell toxicity as a consequence (Citorik et al., 2014). When the target was a chromosomal gene, the lethality was due to irreparable chromosomal damages (Bikard et al., 2014; Citorik et al., 2014). Conversely, no cytotoxicity was observed when CRISPR-Cas9 eliminated a resistance plasmid in bacteria lacking a toxin-antitoxin system (Bikard et al., 2014; Citorik et al., 2014). Plasmid clearance was also achieved in another model strain of *E. coli* in which the *bla_NDM–1_* and *bla_CTX–M–15_* resistance genes were targeted (Yosef et al., 2015). The authors went one step further and simultaneously conferred resistance against the lytic phage T7, thereby providing the re-sensitized bacteria a selective advantage in the presence of the phage. Using this approach, antibiotic treatment along with administration of T7 should robustly oppress the re-emergence of resistant variants.

With the successful reversal of drug resistance in *E. coli* model strains, the consecutive step would be to test clinical isolates. In fact, in our study, plasmid clearance was achieved in the clinical strain of *E. coli* 189A when tackling the *bla*_TEM–__1_ gene present in a low-copy plasmid. Importantly, CRISPR-Cas9 activity led to a simultaneous re-sensitization to several beta-lactam antibiotics including CRO. This is particularly encouraging, given that third generation cephalosporins are “critically important antimicrobials,” which need to be preserved as last resource treatments (Collignon et al., 2016). Re-sensitization effectiveness could be verified *in vivo* by rescuing *E. coli* 189A infected larvae with CRO, confirming the benefit of CRISPR-Cas9 for controlling drug resistant clinical isolates.

Both pSB1C3 vectors harbored by the clinical isolates of *K. variicola* 68AI and *E*. *hormaechei* 4962 demonstrated deletions in the CRISPR-Cas9 locus, and subsequent maintenance of the *bla*_TEM–__1_-carrying low-copy plasmid. This is similar to what has been described by Bikard et al. (2014), who found that survival of *S. aureus* was due to the lack of the Cas9 region after CRISPR-Cas9 delivery, impeding DNA cleavage. Apart from this, plasmid maintenance with the presence of at least one other resistance gene not covered by our CRISPR-Cas9 system might explain why the re-sensitization in *E. hormaechei* 4962 and *K. variicola* 68AI was only partially successful. Given that clinical strains frequently harbor different resistance genes on plasmids (Bennett, 2009; Blair et al., 2015), this signifies that multiple sgRNA for potentially targeting different AMR genes need to be employed, for re-gaining full susceptibility to traditional antibiotics.

Even though the resistance reversal was not completely achieved to the sensitive category in *E. hormaechei* 4962, the intermediate sensitivity may still imply therapeutic success. After all, according to the latest guidelines of the CLSI (2019) and The European Committee on Antimicrobial Susceptibility Testing [EUCAST] (2019), intermediate levels are to be interpreted as persisting clinical efficacy under elevated drug exposure.

Clearly, impediment of re-sensitization can also stem from overexpression of efflux pumps, or from reduction of permeability. Those intrinsic resistance mechanisms contribute to increased levels of resistance against cephalosporins and are typically found in clinical strains of *Enterobacteriaceae* (Blair et al., 2015). Nonetheless, overexpression of efflux pumps or alterations in porins in the clinical isolate of *E. coli*, if having occurred, did not prevent phenotype reversal. Furthermore, despite the versatile challenges imposed by clinical isolates, the interference with the *bla*_TEM–__1_ gene led after all to minor but clear resistance reductions in the other two clinical isolates.

**FIGURE 5:**
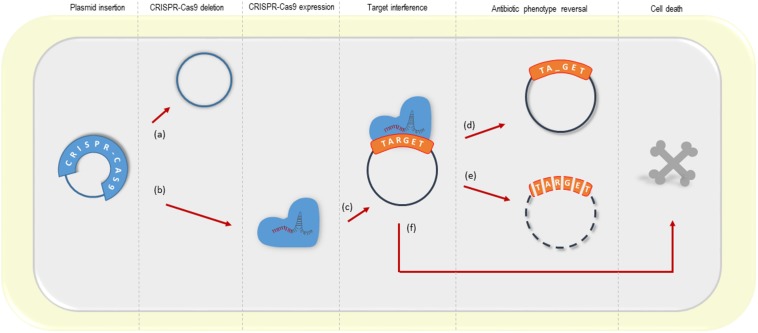
Possible outcomes of the CRISPR-Cas system targeting plasmid resistance genes. (a) deletion of the CRISPR-Cas9 system from its vector (observed in this study; Bikard et al., 2014; Citorik et al., 2014); (b, c) CRISPR-Cas9 expression and target interference; (d) a small amount of plasmid remains into the bacterial cell upon CRISPR-Cas9 interference, but the antibiotic resistance gene from a high-copy plasmid is disrupted (this study); (e) resistance phenotype reversal by plasmid clearance (observed in this study with low and high-copy plasmids; Bikard et al., 2014; Citorik et al., 2014; Yosef et al., 2015; the latter using the CRISPR-Cas type I) (f) cell death driven by the activation of the toxin-antitoxin system (Citorik et al., 2014).

Our findings support multiple aspects of the previous studies and complement the possible outcomes of the CRISPR-Cas9-based interference with plasmid-borne resistance genes, summarized in Figure 5.

When translating the use of the CRISPR-Cas9 system into clinical settings, phages might be an elegant approach for targeted delivery of the system into the bacterial pathogens. Successful application in mice and larvae using bio-engineered phages (Bikard et al., 2014; Citorik et al., 2014) pave the way for a possible future utilization in humans. Alternatively, phage-mediated CRISPR-Cas9 systems could be dispersed on surfaces to serve as preventive disinfection and cleansing procedures along with elaborate selection mechanisms that ensure stability of the CRISPR-Cas systems in bacterial cells, as demonstrated previously (Yosef et al., 2015). Importantly, cases in which CRISPR-Cas9 maintenance in the bacterial cell is intended, resistance genes commonly used as plasmid selective markers should be removed from the CRISPR-Cas9 vector in order to prevent further AMR spread. The possibility to restore sensitivity to traditional antibiotics might be superior to searching for new antimicrobials and potentially could deaccelerate the crisis that we are currently facing with AMR carrying bacteria.

## Data Availability Statement

The genomic dataset generated for this study can be found in the Genbank (accession number: *E. coli* SAMN12872130, *E. hormaechei* SAMN12872875, and *K. variicola* SAMN10216245).

## Ethics Statement

Clinical strain collection was approved by the Human Research Ethics Committee from Universidade Federal de Minas Gerais (Brazil) under the protocol number ETIC 614/08 and written informed consent was obtained from each participant.

## Author Contributions

TT, TM, SS, and H-PH designed the study. TT, NG, and MP performed the research. H-PH, LV, TM, and SS contributed with new reagents. TT, TM, and H-PH analyzed the data. TT, H-PH, SS, and TM wrote the manuscript.

## Conflict of Interest

The authors declare that the research was conducted in the absence of any commercial or financial relationships that could be construed as a potential conflict of interest.

## Footnotes

1 http://parts.igem.org/

## References

[B1] Adams C. P. Brantner V. V. (2006). Estimating the cost of new drug development: is it really $802 Million? *Health Affairs* 25 420–428. 10.1377/hlthaff.25.2.420 16522582

[B2] Bankevich A. Nurk S. Antipov D. Gurevich A. A. Dvorkin M. Kulikov A. S. (2012). SPAdes: a new genome assembly algorithm and its applications to single-cell sequencing. *J. Comput. Biol.* 19 455–477. 10.1089/cmb.2012.0021 22506599 PMC3342519

[B3] Barrios-Camacho H. Aguilar-Vera A. Beltran-Rojel M. Aguilar-Vera E. Duran-Bedolla J. Rodriguez-Medina N. (2019). Molecular epidemiology of *Klebsiella variicola* obtained from different sources. *Sci. Rep.* 9:10610. 10.1038/s41598-019-46998-9 31337792 PMC6650414

[B4] Bennett P. M. (2009). Plasmid encoded antibiotic resistance: acquisition and transfer of antibiotic resistance genes in bacteria: plasmid-encoded antibiotic resistance. *Br. J. Pharmacol.* 153 S347–S357. 10.1038/sj.bjp.0707607 18193080 PMC2268074

[B5] Beyrouthy R. Barets M. Marion E. Dananché C. Dauwalder O. Robin F. (2018). Novel *Enterobacter* lineage as leading cause of nosocomial outbreak involving carbapenemase-producing strains. *Emerg. Infect. Dis.* 24 1505–1515. 10.3201/eid2408.180151 30014838 PMC6056098

[B6] Bikard D. Euler C. W. Jiang W. Nussenzweig P. M. Goldberg G. W. Duportet X. (2014). Exploiting CRISPR-Cas nucleases to produce sequence-specific antimicrobials. *Nat. Biotechnol.* 32 1146–1150. 10.1038/nbt.3043 25282355 PMC4317352

[B7] Blair J. M. A. Webber M. A. Baylay A. J. Ogbolu D. O. Piddock L. J. V. (2015). Molecular mechanisms of antibiotic resistance. *Nat. Rev. Microbiol.* 13 42–51. 10.1038/nrmicro3380 25435309

[B8] Bolger A. M. Lohse M. Usadel B. (2014). Trimmomatic: a flexible trimmer for Illumina sequence data. *Bioinformatics* 30 2114–2120. 10.1093/bioinformatics/btu170 24695404 PMC4103590

[B9] Ceasar S. A. Rajan V. Prykhozhij S. V. Berman J. N. Ignacimuthu S. (2016). Insert, remove or replace: A highly advanced genome editing system using CRISPR/Cas9. *Biochim. Biophys. Acta Mol. Cell Res.* 1863 2333–2344. 10.1016/j.bbamcr.2016.06.009 27350235

[B10] Chan W. Verma C. S. Lane D. P. Gan S. K. (2013). A comparison and optimization of methods and factors affecting the transformation of *Escherichia coli*. *Biosci. Rep.* 33 931–937. 10.1042/BSR20130098 24229075 PMC3860579

[B11] Chayot R. Montagne B. Mazel D. Ricchetti M. (2010). An end-joining repair mechanism in *Escherichia coli*. *Proc. Natl. Acad. Sci. U.S.A.* 107 2141–2146. 10.1073/pnas.0906355107 20133858 PMC2836643

[B12] Chen W. McKenna A. Schreiber J. Haeussler M. Yin Y. Agarwal V. (2019). Massively parallel profiling and predictive modeling of the outcomes of CRISPR/Cas9-mediated double-strand break repair. *Nucleic Acids Res.* 47 7989–8003. 10.1093/nar/gkz487 31165867 PMC6735782

[B13] Citorik R. J. Mimee M. Lu T. K. (2014). Sequence-specific antimicrobials using efficiently delivered RNA-guided nucleases. *Nat. Biotechnol.* 32 1141–1145. 10.1038/nbt.3011 25240928 PMC4237163

[B14] Clinical and Laboratory Standards Institute, Weinstein M. P. (2012). *Methods for Dilution Antimicrobial Susceptibility Tests for Bacteria that Grow Aerobically.* Wayne, PA: Committee for Clinical Laboratory Standards.

[B15] Clinical and Laboratory Standards Institute, (2019). *Performance Standards for Antimicrobial Susceptibility Testing*, 27th Edn Wayne, PA: Committee for Clinical Laboratory Standards.

[B16] Collignon P. C. Conly J. M. Andremont A. McEwen S. A. Aidara-Kane A. (2016). World health organization ranking of antimicrobials according to their importance in human medicine: a critical step for developing risk management strategies to control antimicrobial resistance from food animal production. *Clin. Infect. Dis.* 63 1087–1093. 10.1093/cid/ciw475 27439526

[B17] Gavin L. Lum D. Ng B. Sam C. (2015). Differential transformation efficiencies observed for pUC19 and pBR322 in *E. coli* may be related to calcium chloride concentration. *J. Exp. Microbiol. Immunol.* 20 1–6.

[B18] Gonzales M. F. Brooks T. Pukatzki S. U. Provenzano D. (2013). Rapid Protocol for Preparation of Electrocompetent *Escherichia coli* and *Vibrio cholerae*. *J. Vis. Exp.* 80:50684. 10.3791/50684 24146001 PMC3939052

[B19] Hall J. P. J. Harrison E. (2016). Bacterial evolution: resistance is a numbers game. *Nat. Microbiol.* 1:16235. 10.1038/nmicrobiol.2016.235 27819662

[B20] Harding C. R. Schroeder G. N. Collins J. W. Frankel G. (2013). Use of galleria mellonella as a model organism to study *Legionella pneumophila* infection. *JoVE* 81:e50964. 10.3791/50964 24299965 PMC3923569

[B21] Hoffmann H. Stindl S. Ludwig W. Stumpf A. Mehlen A. Monget D. (2005). Enterobacter hormaechei subsp. oharae subsp. nov., E. hormaechei subsp. hormaechei comb. nov., and E. hormaechei subsp. steigerwaltii subsp. nov., three new subspecies of clinical importance. *J. Clin. Microbiol.* 43 3297–3303. 10.1128/JCM.43.7.3297-3303.2005 16000451 PMC1169129

[B22] Jansen M. Wahida A. Latz S. Krüttgen A. Häfner H. Buhl E. M. (2018). Enhanced antibacterial effect of the novel T4-like bacteriophage KARL-1 in combination with antibiotics against multi-drug resistant *Acinetobacter baumannii*. *Sci. Rep.* 8:14140. 10.1038/s41598-018-32344-y 30237558 PMC6147977

[B23] Kim S. Jeong H. Kim E.-Y. Kim J. F. Lee S. Y. Yoon S. H. (2017). Genomic and transcriptomic landscape of *Escherichia coli* BL21(DE3). *Nucleic Acids Res.* 45 5285–5293. 10.1093/nar/gkx228 28379538 PMC5435950

[B24] Klappenbach J. A. Dunbar J. M. Schmidt T. M. (2000). rRNA operon copy number reflects ecological strategies of bacteria. *Appl. Environ. Microbiol.* 66 1328–1333. 10.1128/AEM.66.4.1328-1333.2000 10742207 PMC91988

[B25] Lachmayr K. L. Kerkhof L. J. DiRienzo A. G. Cavanaugh C. M. Ford T. E. (2009). Quantifying nonspecific TEM -lactamase (blaTEM) genes in a wastewater stream. *Appl. Environ. Microbiol.* 75 203–211. 10.1128/AEM.01254-08 18997031 PMC2612200

[B26] Larionov A. Krause A. Miller W. (2005). A standard curve based method for relative real time PCR data processing. *BMC Bioinformatics* 6:62. 10.1186/1471-2105-6-62 15780134 PMC1274258

[B27] Lim G. Lum D. Ng B. Sam C. (2015). Differential transformation efficiencies observed for pUC19 and pBR322 in *E. coli* may be related to calcium chloride concentration. *J. Exp. Microbiol. Immunol.* 20 1–6.

[B28] Liu W. Xie X. Ma X. Li J. Chen J. Liu Y.-G. (2015). DSDecode: a web-based tool for decoding of sequencing chromatograms for genotyping of targeted mutations. *Mol. Plant* 8 1431–1433. 10.1016/j.molp.2015.05.009 26032088

[B29] Long S. W. Linson S. E. Ojeda Saavedra M. Cantu C. Davis J. J. Brettin T. (2017). Whole-genome sequencing of human clinical *Klebsiella pneumoniae* isolates reveals misidentification and misunderstandings of *Klebsiella pneumoniae*, *Klebsiella variicola*, and *Klebsiella quasipneumoniae*. *mSphere* 2:e00290-17. 10.1128/mSphereDirect.00290-17 28776045 PMC5541162

[B30] O’Neil J. (2016). Tackling Drug-Resistant Infections Globally: Final Report And Recommendations. Available online at: http://amr-review.org (accessed November, 2019).

[B31] Rawat D. Nair D. (2010). Extended-spectrum ß-lactamases in gram negative bacteria. *J. Glob. Infect. Dis.* 2:263. 10.4103/0974-777X.68531 20927289 PMC2946684

[B32] Registry of Standard Biological Parts, (2019). *Plasmid Backbones/Assembly - Parts.igem.org.* Available at: http://parts.igem.org/Plasmid_backbones/Assembly (Accessed September 10, 2018).

[B33] Rodríguez-Medina N. Barrios-Camacho H. Duran-Bedolla J. Garza-Ramos U. (2019). *Klebsiella variicola* : an emerging pathogen in humans. *Emerg. Microbes Infect.* 8 973–988. 10.1080/22221751.2019.1634981 31259664 PMC6609320

[B34] San Millan A. Escudero J. A. Gifford D. R. Mazel D. MacLean R. C. (2016). Multicopy plasmids potentiate the evolution of antibiotic resistance in bacteria. *Nat. Ecol. Evol.* 1:10. 10.1038/s41559-016-0010 28812563

[B35] San Millan A. Santos-Lopez A. Ortega-Huedo R. Bernabe-Balas C. Kennedy S. P. Gonzalez-Zorn B. (2015). Small-plasmid-mediated antibiotic resistance is enhanced by increases in plasmid copy number and bacterial fitness. *Antimicrob. Agents Chemother.* 59 3335–3341. 10.1128/AAC.00235-15 25824216 PMC4432117

[B36] Savard P. Perl T. M. (2012). A call for action: managing the emergence of multidrug-resistant *Enterobacteriaceae* in the acute care settings. *Curr. Opin. Infect. Dis.* 25 371–377. 10.1097/QCO.0b013e3283558c17 22766646

[B37] Schechter L. M. Creely D. P. Garner C. D. Shortridge D. Nguyen H. Chen L. (2018). Extensive gene amplification as a mechanism for piperacillin-tazobactam resistance in *Escherichia coli*. *mBio* 9:e00583-18. 10.1128/mBio.00583-18 29691340 PMC5915731

[B38] Studier F. W. Moffatt B. A. (1986). Use of bacteriophage T7 RNA polymerase to direct selective high-level expression of cloned genes. *J. Mol. Biol.* 189 113–130. 10.1016/0022-2836(86)90385-2 3537305

[B39] The European Committee on Antimicrobial Susceptibility Testing [EUCAST], (2019). *New definitions of S, I and R.* Available online at: http://www.eucast.org/newsiandr/ (accessed July, 2019).

[B40] Van den Bergh B. Fauvart M. Michiels J. (2017). Formation, physiology, ecology, evolution and clinical importance of bacterial persisters. *FEMS Microbiol. Rev.* 41 219–251. 10.1093/femsre/fux001 28333307

[B41] Van Norman G. A. (2016). Drugs, devices, and the FDA: Part 1. *JACC* 1 170–179. 10.1016/j.jacbts.2016.03.002 30167510 PMC6113160

[B42] Větrovský T. Baldrian P. (2013). The variability of the 16S rRNA gene in bacterial genomes and its consequences for bacterial community analyses. *PLoS One* 8 e57923. 10.1371/journal.pone.0057923 23460914 PMC3583900

[B43] Vianna M. E. Holtgraewe S. Seyfarth I. Conrads G. Horz H. P. (2008). Quantitative analysis of three hydrogenotrophic microbial groups, methanogenic archaea, sulfate-reducing bacteria, and acetogenic bacteria, within plaque biofilms associated with human periodontal disease. *J. Bacteriol.* 190 3779–3785. 10.1128/JB.01861-07 18326571 PMC2394984

[B44] Wood T. K. Knabel S. J. Kwan B. W. (2013). Bacterial persister cell formation and dormancy. *Appl. Environ. Microbiol.* 79 7116–7121. 10.1128/AEM.02636-13 24038684 PMC3837759

[B45] Yang S. Sleight S. C. Sauro H. M. (2013). Rationally designed bidirectional promoter improves the evolutionary stability of synthetic genetic circuits. *Nucleic Acids Res.* 41:e33. 10.1093/nar/gks972 23093602 PMC3592475

[B46] Yosef I. Manor M. Kiro R. Qimron U. (2015). Temperate and lytic bacteriophages programmed to sensitize and kill antibiotic-resistant bacteria. *Proc. Natl. Acad. Sci. U.S.A.* 112 7267–7272. 10.1073/pnas.1500107112 26060300 PMC4466736

[B47] Zankari E. Hasman H. Cosentino S. Vestergaard M. Rasmussen S. Lund O. (2012). Identification of acquired antimicrobial resistance genes. *J. Antimicrob. Chemother.* 67 2640–2644. 10.1093/jac/dks261 22782487 PMC3468078

