# Supplementary material for: Exploring the Potential of CRISPR-Cas9 Under Challenging Conditions: Facing High-Copy Plasmids and Counteracting Beta-Lactam Resistance in Clinical Strains of Enterobacteriaceae
Source: Front Microbiol. 2020 Apr 30;11:578. doi: 10.3389/fmicb.2020.00578 (PMC7203346; doi:10.3389/fmicb.2020.00578)
Supplement: Supplementary file 1 [file Data_Sheet_1.PDF]

Supplementary material:

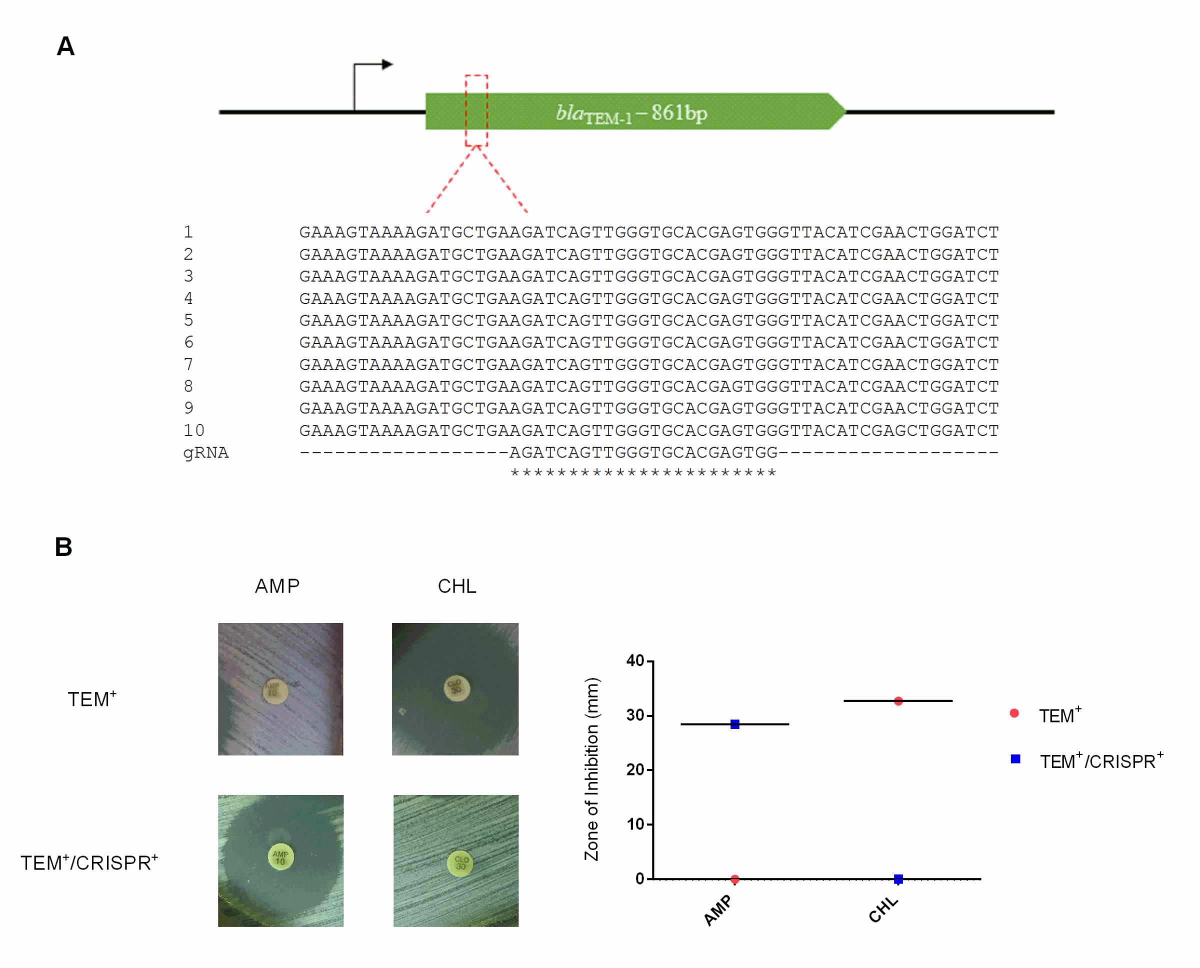

Fig. S1. gRNA design and CRISPR-Cas9 resistance reversal in a model strain of *E. coli*. **(A)**: Alignment of representative *bla*$_{\text{TEM-1}}$ sequences retrieved from Genbank and position of the designed gRNA. The selected bacteria and the sequence accession number of the plasmid/gene are: 1. *E. coli* NC_013728.1; 2. *Escherichia coli* EU418920.2; 3. *E. cloacae* NC_019368.1; 4. *E. coli* NC_022885.1; 5. *Klebsiella pneumoniae strain Kp145/11* KX118608.1; 6. *K. pneumoniae* FJ560503.1; 7. *K. pneumoniae* KJ663712.1; 8. *K. pneumoniae* EF035581.1; 9. *Enterobacter* sp. NC_015515.1; 10. *E. coli* AF188200.1. **(B)**: Disk diffusion susceptibility test on *E. coli* BL21 grown on Mueller-Hinton agar with measurement of inhibition zones showing re-sensitization to ampicillin (AMP), along with acquired resistance against chloramphenicol (CHL), co-mediated via the CRISPR-Cas9 plasmid. TEM^{+}: *E. coli* with the *bla*$_{\text{TEM-1}}$ harbouring

plasmid; TEM+/CRISPR+: *E. coli* with the *bla*$_{\text{TEM-1}}$ harbouring plasmid and the CRISPR-Cas9 harbouring plasmid.

A

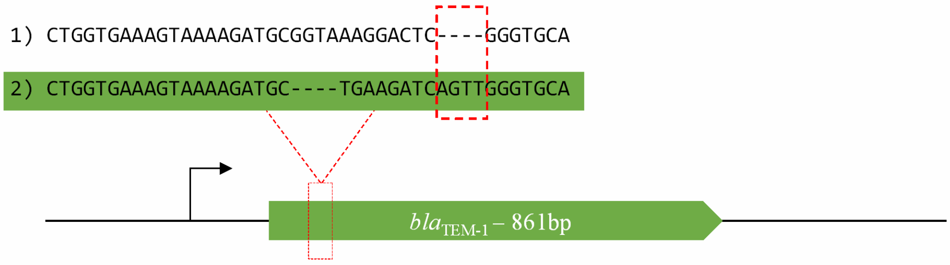

B

	AMP	CRO	SAM	AMC	CAZ	CFZ	FOX	CXM	FEP	ATM	CTX	CHL
E. coli 189A^{WT}	0	12.46	16.79	20.83	23.13	0	21.11	0	21.54	21.3	15.86	26.53
	0	11.29	23.85	24.08	23.3	0	23.25	0	28.86	20.44	11.55	24.34
E. coli 189A$^{\text{CRISPR+}}$	18.33	27.49	21.39	22.52	25.75	20.84	22.56	20.71	29.23	29.02	26.32	0
	18.84	27.38	19.87	23.79	22.64	19.37	24.75	20.45	28.58	27.89	26.64	0
E. hormaechei 4962^{WT}	0	5.24	0	0	9.75	0	7.28	0	20.20	7.63	0	22.08
	0	6.67	0	0	10.77	0	7.03	0	20.50	8.84	0	22.50
E. hormaechei 4962$^{\text{CRISPR+}}$	0	12.09	0	0	12.96	0	7.31	0	19.81	17.57	10.34	0
	0	13.01	0	0	12.26	0	8.73	0	23.73	20.17	11.97	0
K. variicola 68AI^{WT}	0	7.13	12.38	22.3	16.93	0	20.87	0	16.08	17.08	7.93	22.83
	0	8.85	13.17	17.17	16.31	0	24.95	0	17.43	18.69	10.88	22.58
K. variicola 68AI$^{\text{CRISPR+}}$	0	9.20	13.27	16.85	17.7	0	23.10	0	18.59	18.53	11.03	0
	0	8.97	10.83	17.87	19.57	0	23.58	0	18.52	17.58	11.25	0

Fig. S2: Sequence alterations in the *bla*$_{\text{TEM-1}}$ gene after CRISPR-Cas9 transformation into *E. coli* BL21 and the spectrum of resistance reduction in clinical isolates. **(A)**: A representative TEM+/CRISPR+ colony sequence analysed by the DSDecodeM showing the presence of an allele containing deletions in the gRNA sequence, highlighted in red (sequence 1). Sequence number 2 represents the control without interference of CRISPR-Cas9. **(B)**: Schematic representation of the disk diffusion susceptibility results of all the tested antibiotics in three Enterobacteriaceae clinical strains. Values refer to the measured diameter of the inhibition zones. Green, yellow and red boxes represent sensitive, intermediate (or dose dependant) and

resistant category, respectively, according to CLSI classification. CRO: ceftriaxone; AMP: ampicillin; CTX: cefotaxime; CXM: cefuroxime; CFZ: cefazolin; CHL: chloramphenicol; ATM: aztreonam; CAZ: ceftazidime; FEP: cefepime; SAM: ampicillin/sulbactam; AMC: amoxicillin/clavulanate; FOX: cefoxitin.

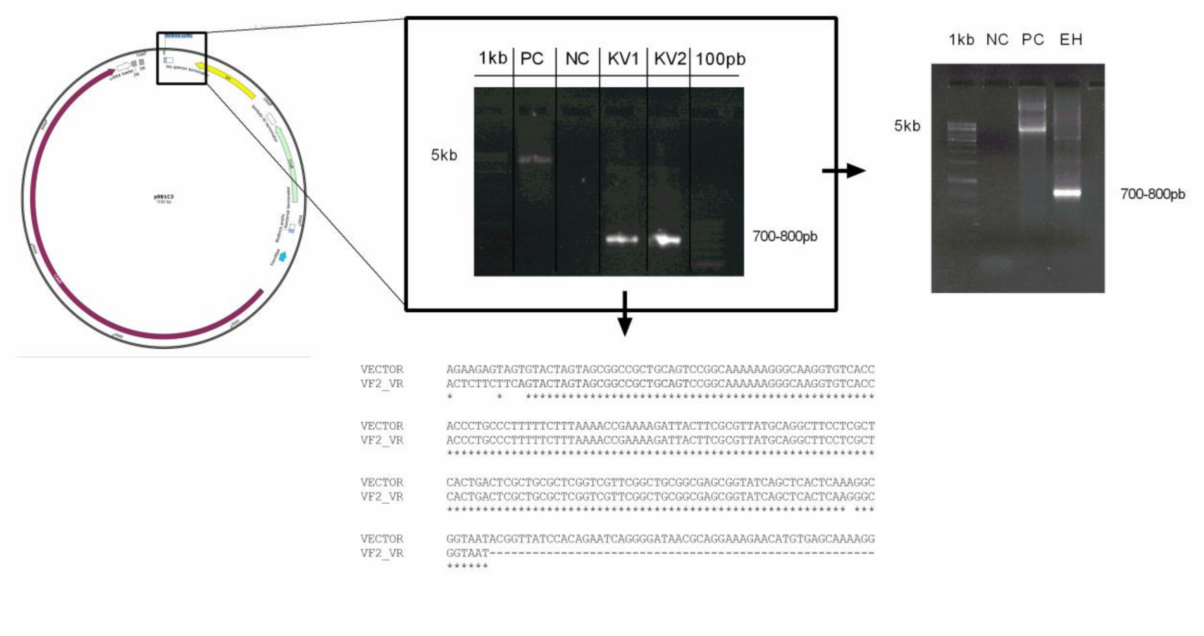

Fig. S3: Map of the plasmid pSB1C3 and PCR-based detection of the CRISPR-Cas 9 system. After transformation of the plasmid in a clinical isolate of *K. variicola* 68AI strain, PCR amplification led to a fragment of only 700-800 bp in size (instead of the targeted full size amplicon of around 5 kb). Sequence analysis and subsequent alignment revealed that this PCR product represented only the vector backbone, without the CRISPR-Cas9 system suggesting degradation within the bacterial cell. PC: Positive control; NC: Negative control; KP1 and KP2: *K. variicola* 68AI$^{CRISPR+}$ (duplicate PCR assay); EH: *E. hormaechei* 4962.
