# Supplementary material for: Exploring the Potential of CRISPR-Cas9 Under Challenging Conditions: Facing High-Copy Plasmids and Counteracting Beta-Lactam Resistance in Clinical Strains of Enterobacteriaceae
Source: Front Microbiol. 2020 Apr 30;11:578. doi: 10.3389/fmicb.2020.00578 (PMC7203346; doi:10.3389/fmicb.2020.00578)
Supplement: Supplementary file 2 [file Data_Sheet_2.PDF]

Tables:

Supplementary Table S1: Primers used in this study

Primer denomination	Sequence (5`-3`)	Conditions	Reference
**Cas9F**	ACGCATTGATTTGAGTCAGC	95°C, 3 s, 40x: 95°C, 5s; 55°C, 30s.	This study
**Cas9R**	GACCTTTGAGCTTCCGAGAC		This study
**16SF**	GCTCAGATTGAACGCTGG		Zucol *et al.*, 2006
**16SR**	TACTGCTGCCTCCCGTA		Zucol *et al.*, 2006
**BLATEMF**	ATGAGTATTCAACATTTCCG	94°C, 4m, 30x: 94°C, 1m; 47°C, 30s; 72°C, 1m, and 72°C, 4m.	This study
**BLATEMR**	TTACCAATGCTTAATCAGTG		This study
**TEMF**	GTGCACGAGTGGGTTACATC	95°C, 3 s, 40x: 95°C, 5s; 55°C, 30s.	This study
**TEMR**	AGAAGTAAGTTGGCCGCAGT		This study
**VF2**	TGCCACCTGACGTCTAAGAA	94°C, 2m, 30x: 94°C, 30s; 50°C, 30s; 72°C, 6m, 72°C, 7m.	Registry of Standard Biological Part
**VR**	ATTACCGCCTTTGAGTGAGC		Registry of Standard Biological Part

Supplementary Table S2 – Clinical strains used in this study

Strain Number	Strains	Description	Reference
**5**	***E. coli* 189A^{WT}**	Clinical strain isolated from a bacteraemia patient	This study
**6**	***E. coli* 189A$^{CRISPR+}$**	Strain #5, treated with CRISPR-Cas9	This study
**7**	***E. cloacae* 4962^{WT}**	Clinical strain isolated from a bacteraemia patient	This study
**8**	***E. cloacae* 4962$^{CRISPR+}$**	Strain #7, treated with CRISPR-Cas9	This study
**9**	***K. variicola* 68AI^{WT}**	Clinical strain isolated from a paediatric bacteraemia patient	This study
**10**	***K. variicola* 68AI$^{CRISPR+}$**	Strain #9, treated with CRISPR-Cas9	This study

References:

Studier FW, Moffatt BA (1986) Use of bacteriophage T7 RNA polymerase to direct selective high-level expression of cloned genes. *J Mol Biol* 189(1):113–130.

Kim S, et al. (2017) Genomic and transcriptomic landscape of Escherichia coli BL21(DE3). *Nucleic Acids Res* 45(9):5285–5293.

Zucol F, et al. (2006) Real-Time Quantitative Broad-Range PCR Assay for Detection of the 16S rRNA Gene Followed by Sequencing for Species Identification. *Journal of Clinical Microbiology* 44(8):2750–2759.

Registry of Standard Biological Part. http://parts.igem.org/ . Accessed in April 2019.
